# Patterns of Musculoskeletal Involvement in Hidradenitis Suppurativa: A Pilot Study

**DOI:** 10.7759/cureus.106996

**Published:** 2026-04-13

**Authors:** Yossra Suliman, Enas Attia

**Affiliations:** 1 Rheumatology and Rehabilitation, Assiut University, Assiut, EGY; 2 Rheumatology, Ain Al Khaleej Hospital, Abu Dhabi, ARE; 3 Dermatology, Ain Shams University, Cairo, EGY; 4 Dermatology, Ain Al Khaleej Hospital, Abu Dhabi, ARE

**Keywords:** arthritis, enthesitis, hidradenitis suppurativa, il-17 inhibitors, metabolic syndrome, musculoskeletal, secukinumab, tnf inhibitors

## Abstract

Background: Despite increasing evidence associating hidradenitis suppurativa (HS) with peripheral arthritis, enthesitis, and axial inflammation, its clinical manifestations and optimal management approaches remain insufficiently studied.

Objectives: This pilot study was conducted to characterize patterns of musculoskeletal (MSK) involvement in HS patients, assess disease severity and related metabolic comorbidities, and determine the impact of biologic therapy on symptom control.

Methods: A retrospective observational pilot study was conducted involving patients with HS presenting with concurrent MSK symptoms. Clinical data were collected on disease severity, patterns of joint involvement, presence of enthesitis, metabolic parameters, and treatment outcomes. Patients were stratified into axial, peripheral, and mixed MSK phenotypes. Laboratory markers, including glycated hemoglobin (HbA1c), were evaluated to explore potential metabolic associations. Additionally, treatment regimens, comprising nonsteroidal anti-inflammatory drugs (NSAIDs), antibiotics, and biologic therapies such as tumor necrosis factor (TNF) inhibitors and interleukin-17 (IL-17) inhibitors, were reviewed.

Results: Eight patients with HS and concurrent MSK involvement were identified. Among them, 62.5% presented with peripheral arthritis, 12.5% with axial involvement, and 25% with mixed phenotypes. All patients were classified as Hurley stage II or III, with no clear association between disease severity and the pattern of MSK involvement.

Enthesitis emerged as a prominent clinical feature, affecting 75% of our patients, most frequently affecting the Achilles tendon and plantar fascia. Metabolic dysfunction was also highly prevalent, including obesity in 62.5% of patients, fatty liver disease in 75%, and elevated HbA1c levels in 75%.

NSAIDs provided partial short-term symptomatic relief. In contrast, biologic therapies, particularly IL-17 inhibitor (secukinumab), demonstrated greater efficacy in controlling both MSK and cutaneous manifestations. Notably, patients who were switched from TNF inhibitors to IL-17 inhibitors reported improved clinical outcomes.

Conclusions: HS-associated MSK involvement manifests in diverse clinical patterns and is often associated with metabolic comorbidities. Inhibition of IL-17 has emerged as a promising therapeutic approach, particularly for patients who exhibit inadequate responses to TNF inhibitors. However, larger-scale screening and long-term prospective studies are required to better characterize MSK prevalence, onset, clinical patterns, and relationship with cutaneous disease severity and activity, as well as to establish optimal management strategies for HS and HS-associated arthritis.

## Introduction

Hidradenitis suppurativa (HS) is a chronic, relapsing inflammatory skin disorder characterized by painful suppurative nodules, abscesses, and the formation of sinus tracts. The associated pain and persistent suppuration often result in reduced physical activity, psychological distress, and a markedly diminished quality of life [1].

Beyond its cutaneous manifestations, HS has increasingly been associated with systemic inflammatory comorbidities, including musculoskeletal (MSK) involvement. Although this association remains underrecognized, emerging evidence indicates a considerable burden of peripheral arthritis, enthesitis, and axial inflammation among affected individuals. These manifestations frequently contribute to ongoing pain and functional impairment, underscoring the need for more comprehensive and effective management strategies [2].

Biologic therapies, particularly tumor necrosis factor (TNF) inhibitors and interleukin-17 (IL-17) inhibitors, have demonstrated promising efficacy in addressing both the dermatologic and MSK features of the disease [3]. However, their role in HS and HS-associated arthritis warrants further investigation.

In this retrospective pilot study, we aimed to characterize the patterns of MSK involvement in patients with HS, as well as to evaluate disease severity, the prevalence of metabolic syndrome (MetS), and the impact of biologic therapy within our center.

## Materials and methods

This retrospective, descriptive, observational pilot study included patients diagnosed with HS and concurrent MSK symptoms who attended our center between May 1, 2022, and April 30, 2024.

Medical records from outpatient visits in both the rheumatology and dermatology units were reviewed. Patients with concomitant diagnoses of HS and arthritis and/or enthesitis were included. Demographic and clinical data were collected, including cutaneous disease severity, patterns of joint involvement and enthesitis, metabolic comorbidities, and management strategies.

Disease severity of HS was assessed using the Hurley staging system, as described by Saunte et al. [4]. Patients with incomplete data or with other clinically defined rheumatologic diseases were excluded.

A total of eight patients met the inclusion criteria and were included in the analysis. Patients were categorized into axial, peripheral, or mixed MSK phenotypes according to Rudwaleit et al. (2011) [5].

Variables analyzed included age, sex, and body mass index (BMI) as a marker of obesity, as well as glycated hemoglobin (HbA1c) levels and the presence of fatty liver.

Treatment regimens, including nonsteroidal anti-inflammatory drugs (NSAIDs), antibiotics, and biologic therapies (TNF and IL-17 inhibitors), were reviewed to evaluate their effectiveness in controlling MSK symptoms.

Given the small sample size, only descriptive statistics (frequencies and percentages) were used.

The study was approved by our hospital's Institutional Review Board (IRB) (AKH/2024002 #7), with a waiver of informed consent due to its retrospective design and reliance on anonymized medical record data without direct patient interaction. No identifying patient information was included in the study.

## Results

Skin disease severity assessment

Cutaneous disease severity was evaluated using the Hurley staging system, as described by Saunte et al. [4]. Stage I is characterized by transient, non-scarring inflammatory lesions. Stage II is defined by recurrent abscesses with sinus tract (tunnel) formation and scarring, presenting as single or multiple lesions separated by areas of unaffected skin. Stage III is marked by diffuse or coalescent lesions with extensive tunnel formation, scarring, and persistent inflammation. All patients in our cohort were classified as Hurley stage II-III (Table 1).

**Table 1:** Cutaneous disease severity and patterns of musculoskeletal involvement and associated comorbidities with treatment response in studied patients. MSK: musculoskeletal; MCP: metacarpophalangeal; MTP: metatarsophalangeal; PIP: proximal interphalangeal; SI: sacroiliac; ALT: alanine aminotransferase; AST: aspartate aminotransferase; HbA1c - glycosylated hemoglobin; NSAID: nonsteroidal anti-inflammatory drugs.

Patient ID	Sex	Age	Hurley stage	MSK pattern	Joints involved	Fatty liver	HbA1c	BMI	Initial treatment	Biologics	Response	Notes
1	M	39	II	Peripheral	Bilateral shoulders, elbows, left wrist, ankles, left knee (minimal effusion)	Fatty liver, ALT	8	32	NSAID + antibiotics	Adalimumab (secondary failure), secukinumab	Controlled	High cholesterol
2	M	30	III	Peripheral	Bilateral wrist MCPs and PIPs swollen, tender right ankle, MTPs	Fatty liver, ALT	5.8	34	NSAID + antibiotics	-	Lost to follow-up	High cholesterol
3	M	21	III	Axial	Bilateral sacroiliitis (left > right), bilateral hip arthritis	Raised ALT	5.8	29	NSAID	Secukinumab	Controlled	High uric acid
4	F	32	III	Peripheral, intermittent	Bilateral knee	Fatty liver	6.6	27	NSAID	-	Lost to follow-up	
5	F	38	III	Peripheral + axial	Bilateral shoulders, sternoclavicular joint, bilateral wrists, SI joints, knees, ankles	Normal	5	28	NSAID	Secukinumab (for skin)	Controlled	Scalp psoriasis
6	M	19	III	Peripheral	Bilateral shoulders, elbows, right hip, right knee, ankle	Raised ALT & AST	5.6	30	Surgery, NSAID	Adalimumab (primary failure for skin), secukinumab (for skin)	Controlled	
7	F	27	II	Peripheral	Tender right shoulder, Achilles tendon, bilateral ankles, MTPs, subtalar joints	Normal	6.4	32	NSAID	-	Controlled	Tirzepatide
8	M	44	II	Axial + peripheral	Tender SI joints, bilateral ankles, MTPs, knees, shoulders, elbows, wrists	Fatty liver	6.5	34	NSAID	Secukinumab	Controlled	High uric acid

Musculoskeletal involvement

A total of eight patients with MSK involvement were included in the analysis. The patterns of MSK involvement were categorized as follows: five patients (62.5%) exhibited a peripheral arthritis pattern, one (12.5%) had axial disease, and two (25%) demonstrated a combination of both axial and peripheral involvement.

Peripheral arthritis predominantly affected the shoulders, wrists, knees, and ankles, and was often associated with intermittent swelling and tenderness. Enthesitis was observed in six cases (75%), most commonly involving the Achilles tendon and plantar fascia. Patients with axial involvement presented with sacroiliitis, with one case demonstrating a marked lateral predominance (left > right) (Table 1).

Comorbidities

Metabolic abnormalities were highly prevalent within this cohort. Obesity (BMI ≥30) was observed in five patients (62.5%). Glycemic abnormalities were also common, with three patients exhibiting prediabetic HbA1c levels (5.7-6.4%) and three patients meeting diagnostic criteria for diabetes (HbA1c ≥6.5%).

Additionally, six patients (75%) demonstrated either increased liver echogenicity on ultrasound or elevated liver enzyme levels, both indicative of hepatic steatosis. Collectively, these findings highlight a strong association between HS-associated arthritis and MetS.

Other comorbid conditions included psoriasis in one patient, hypercholesterolemia in two patients, and hyperuricemia in two patients (Table 1).

Response to treatment

NSAIDs provided short-term symptomatic partial relief of MSK symptoms but were generally insufficient for long-term disease control. Two patients initially treated with the TNF inhibitor adalimumab experienced treatment failure and were subsequently switched to the IL-17 inhibitor secukinumab, resulting in improved MSK and cutaneous outcomes. Another patient with axial involvement achieved significant symptom relief with secukinumab (300 mg every four weeks). Two patients with skin-predominant disease and intermittent joint symptoms maintained good control of MSK disease on NSAIDs alone. In contrast, two patients with uncontrolled skin-predominant disease required secukinumab dose escalation to 300 mg every two weeks for optimal management. Unfortunately, two patients were lost to follow-up.

## Discussion

Our study underscores the broad spectrum of MSK involvement in HS, ranging from peripheral arthritis to axial inflammation with sacroiliitis. Enthesitis emerged as a prominent manifestation, indicating a significant overlap with spondyloarthritis (SpA). Nevertheless, given the high BMI in this cohort, plantar fasciitis of mechanical origin cannot be ruled out. The high prevalence of metabolic comorbidities, including fatty liver disease, diabetes, and obesity, raises important questions regarding shared inflammatory pathways between HS and MetS. IL-17 inhibition appears to be a promising therapeutic option for HS patients with MSK involvement, particularly in those who do not respond to TNF inhibitors. Nevertheless, larger studies are needed to establish the optimal biologic treatment strategy for this patient population.

Our cohort highlights that HS is not merely a localized skin disorder but is associated with a range of systemic inflammatory conditions, consistent with prior research [2]. This suggests that moderate-to-severe HS may trigger an exaggerated inflammatory response, leading to multi-organ involvement. Reported systemic associations include cerebrocardiovascular complications, renal impairment, liver disease, alopecia areata, psoriasis, inflammatory eye disorders, inflammatory bowel disease, hypothyroidism, and depression [6].

Regarding MSK manifestations, multicenter studies have reported that approximately 28.2% of HS patients display features of SpA, substantially higher than the 1% prevalence observed in the general population [7,8]. Additionally, a cross-sectional study found that 24% of HS patients experienced prolonged morning stiffness, while 16% had pre-existing arthritis [9].

Another meta-analysis examining the relationship between HS and inflammatory arthritis reported a significant association between the two conditions. Patients with HS were found to have a higher risk of developing inflammatory arthritis compared to individuals without HS. Specifically, HS patients exhibited an overall 3.44-fold increased risk of inflammatory arthritis. The analysis further identified that HS is associated with SpA, showing a 2.10-fold increased risk, and with ankylosing spondylitis (AS), with a 1.89-fold increased risk. A significant association was also observed with rheumatoid arthritis (RA), demonstrating a 1.96-fold increased risk [10]. These findings underscore the importance of screening HS patients for MSK involvement and closely monitoring those with a history of current or previous joint pain. Larger multicenter studies are warranted to clarify the onset, frequency, and patterns of MSK involvement in HS, as well as its correlation with disease severity and activity.

Regarding MetS, several studies have investigated the relationship between HS and MetS. Luo et al. (2024) [11] employed an advanced methodology, conducting a bidirectional two‐sample Mendelian randomization (MR) analysis using summary statistics from genome‐wide association studies (GWAS) of MetS and HS. Their MR analysis demonstrated that MetS causally increased the risk of HS development (inverse-variance weighting (IVW) odds ratio (OR): 1.428; 95% CI: 1.193-1.710; p < 0.001), with consistent support from sensitivity analyses. Conversely, HS did not appear to causally affect MetS risk (IVW OR: 1.008; 95% CI: 0.988-1.028; p = 0.438), indicating that while MetS increases the likelihood of developing HS, the reverse does not hold. This provides a plausible explanation for the high prevalence of MetS observed in our cohort. These findings underscore the importance of addressing MetS, as effective management may prevent or slow HS progression. In our cohort, one patient achieved disease control with NSAIDs and tirzepatide. Interestingly, glucagon-like peptide-1 (GLP-1) agonists have also been reported to exert beneficial effects on immune-mediated skin diseases [12].

HS patients are associated with a significantly increased risk of liver diseases, particularly non-alcoholic fatty liver disease [13]. This underscores the importance of considering such comorbidities when designing screening and management strategies.

One of our patients presented with concomitant psoriasis. Notably, patients with psoriasis have an 80% higher likelihood of developing HS [14].

Systemic therapies, particularly biologics, can effectively mitigate systemic dysfunction driven by HS-triggered inflammatory responses, highlighting the importance of controlling cutaneous inflammation in HS patients [15]. Regarding the management of MSK involvement, intermittent polyarthritis generally responds well to NSAIDs as short-term symptomatic treatments, which are frequently used to manage pain associated with both HS and its arthritic manifestations [16]. In cases of persistent or nonresponsive arthritis, newer therapies, such as TNF inhibitors and IL-17 inhibitors, have shown efficacy in controlling both skin and joint involvement. Secukinumab, in particular, has demonstrated positive outcomes in phase III trials (SUNSHINE and SUNRISE), leading to its approval for HS treatment [17]. In a multicenter study, 77.8% of patients achieved HS clinical response (HiSCR) after 52 weeks of secukinumab therapy [18]. IL-17 inhibitors have demonstrated safety profiles comparable to other biologics, with adverse events predominantly mild [19,20]. These findings align with our study, where biologic therapy was necessary in certain cases to control both cutaneous and MSK manifestations, for further head-to-head studies. Despite the fact that IL-17 inhibitors could be superior in controlling cutaneous and MSK disease, it is of note that HS has a well-established association with inflammatory bowel disease (IBD), where IL-17 inhibitors are contraindicated in active disease [21].

We acknowledge the limitations of this study, including the small sample size, absence of control groups, absence of validated clinical diagnostic criteria, and its retrospective design. Additionally, a uniform radiologic diagnostic approach was lacking. Only patients with sacroiliitis had MRI-confirmed disease, whereas in patients with peripheral joint involvement, clinical examination findings were relied upon due to the retrospective nature of the study. Furthermore, comprehensive metabolic screening and appropriate confirmatory tests, as well as HLA-B27 typing, were lacking. Future multicenter, prospective, case-controlled studies, implementing standardized clinical assessment tools, are warranted to better define the onset, frequency, and pattern of MSK involvement in HS and to correlate these findings with cutaneous disease severity and activity, as well as clinical outcomes, using validated scores for both cutaneous and MSK disease.

## Conclusions

HS-associated MSK disease is an underrecognized yet clinically significant condition. Our findings underscore the broad spectrum of both peripheral and axial joint involvement. MetS is highly prevalent among HS patients with MSK manifestations. Biologic therapies, particularly IL-17 inhibitors, probably show potential in addressing both cutaneous and joint symptoms, warranting further investigation. Larger prospective cohort studies are needed to clarify the temporal relationship between MetS, MSK involvement, and HS, as well as to assess how controlling inflammation through treatment may influence metabolic abnormalities. Given the higher prevalence of MetS, its individual components, and MSK abnormalities in HS patients, routine screening for early detection is essential to optimize disease management.
